# Neural Substrates of Attentional Control During Emotional Processing: Evidence From rTMS–fMRI Targeting the Frontal Eye Field

**DOI:** 10.1002/hbm.70535

**Published:** 2026-04-28

**Authors:** Jennifer Malsert, Vincent Rochas, Tonia Rihs, Swann Pichon, Patrik Vuilleumier

**Affiliations:** ^1^ Department of Psychology and Educational Sciences University of Geneva Geneva Switzerland; ^2^ University of Teacher Education of the State of Vaud Lausanne Switzerland; ^3^ Laboratory for Behavioral Neurology and Imaging of Cognition, Department of Neuroscience University of Geneva Geneva Switzerland; ^4^ M/EEG & Neuromod Platform, Campus Biotech Geneva Foundation Geneva Switzerland; ^5^ Functional Brain Mapping Laboratory, Department of Basic Neurosciences University of Geneva Geneva Switzerland; ^6^ Federal Statistical Office (FSO) Neuchâtel Switzerland; ^7^ Geneva School of Health Sciences, HES‐SO University of Applied Sciences and Arts Western Switzerland Geneva Switzerland; ^8^ Swiss Centre for Affective Sciences, Campus Biotech, University of Geneva Geneva Switzerland; ^9^ Neurology Department University Hospital of Geneva Geneva Switzerland

**Keywords:** amygdala, attentional network, continuous theta‐burst stimulation (cTBS), emotional processing, fearful faces, functional magnetic resonance imaging (fMRI), fusiform face area (FFA)

## Abstract

Past research has provided conflicting evidence concerning whether emotional processing in the amygdala arises independent of selective attention to threat‐related stimuli or instead depends on attentional resources and top‐down voluntary control. Here, we combine repetitive transcranial magnetic stimulation (rTMS) targeting the right frontal eye field (FEF) with functional magnetic resonance imaging (fMRI) to examine how perturbing top‐down attentional control is associated with changes in neural responses to emotional stimuli in visual cortex and amygdala. Participants performed a matching task in which they had to judge whether task‐relevant image pairs were similar or different while ignoring task‐irrelevant pairs. On each trial, one pair showed houses and the other pair displayed either neutral or fearful faces. The task was performed in two sessions following either rTMS or no TMS, in counterbalanced order. Behavioral results revealed that right FEF perturbation selectively slowed responses to neutral but not fearful faces. ROI analyses revealed selective changes in fusiform face area (FFA) responses to neutral faces following FEF rTMS, while responses to fearful faces were relatively preserved; in parallel, amygdala responses to fearful faces remained intact or showed increased activation. A control group undergoing the same protocol with rTMS applied to the vertex (VTX) showed no significant changes in behavioral performance or neural activation patterns. Together, these findings suggest that neural responses to emotionally salient stimuli may be less dependent on top‐down attentional modulation than responses to neutral stimuli, consistent with models proposing partially distinct contributions of attentional and emotional processing networks.

## Graphical abstract

Using rTMS over the frontal eye field combined with fMRI, we demonstrate that disrupting attentional control slows responses and reduces cortical activity for neutral faces while sparing or amplifying amygdala responses to fearful faces, highlighting a disinhibitory effect of TMS on top‐down control.

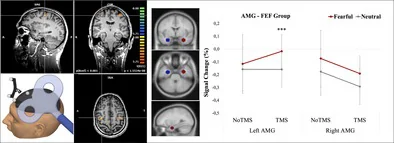

## 1 Introduction

The capacity to detect threats rapidly is essential for adaptive behavior and survival. However, it remains debated whether this capacity arises from an automatic/reflexive neurophysiological prioritization of emotionally salient stimuli, or whether it is gated by brain regions responsible for the voluntary allocation of attention and the availability of cognitive resources. The goal of this study is to address this issue by combining neurostimulation and BOLD imaging techniques to disentangle the reciprocal interactions between attentional control and emotion‐processing networks. Although multiple perspectives have been employed in the literature to investigate the modulation of visual perception by attentional control mechanisms and their interaction with emotional processing, to the best of our knowledge, no study has used a transient interference approach with TMS to address this issue.

Since the pioneering work of Posner in the 1980s, it has been well established that attention can be directed independently of eye movements through spatial orienting mechanisms (Posner 1980), and that it relies on specific functional brain systems (Posner and Petersen 1990; Kastner et al. 1999). Attention allows for selective perception and action toward behaviorally relevant stimuli, while ignoring irrelevant information, thus constituting a fundamental cognitive process that operates through a dynamic balance between goal‐directed activation and inhibitory control (Lupiáñez et al. 2006). This balance is flexibly adjusted according to contextual and environmental demands (Van Moorselaar and Slagter 2020; Forte et al. 2024; Frisch et al. 2015). At the brain level, attentional mechanisms rely on a distributed network involving frontal and parietal cortical areas, including key nodes such as the frontal eye field (FEF) and the intraparietal sulcus (IPS) in both hemispheres (Vossel et al. 2012). Importantly, this network also encompasses subcortical and temporal regions, including the superior colliculus (Krauzlis et al. 2013), pulvinar, and temporal cortex (Ramezanpour and Fallah 2022), as well as frontal regions such as the inferior frontal junction, which jointly contribute to attentional orienting, salience processing, and the integration of sensory with cognitive and motivational signals (Baldauf and Desimone 2014). Together, fronto‐parietal cortical areas and subcortical areas exert attentional control on perception by modulating neural responses in lower‐level sensory regions, including category‐selective regions of the ventral visual cortex, by enhancing activity to behaviorally relevant stimuli and suppressing irrelevant distractors (Kastner and Ungerleider 2000; Vuilleumier and Driver 2007). Notably, the fusiform face area (FFA), a key region for face perception, activates much more strongly when attention is focused on a face rather than on another object, even when stimuli are adjacent or overlap (Wojciulik et al. 1998; O'Craven et al. 1999; Vuilleumier et al. 2001).

Various factors may influence attentional orienting. In particular, the occurrence of salient stimuli—especially those with emotional relevance—can capture processing resources without voluntary cognitive control, partly implicating subcortical pathways outside the regulatory control of cortical fronto‐parietal areas (Tamietto and de Gelder 2010; Pourtois et al. 2013). For instance, behavioral studies have shown that task‐irrelevant emotional stimuli tend to escape or impair voluntary attentional performance, leading to slower reaction times and higher error rates in tasks such as Go/No‐Go (Contreras et al. 2013) or Flanker paradigms (Cohen et al. 2013) in the presence of emotional distractors.

In line with these findings, functional neuroimaging studies have reported that a key neural substrate for these effects involves the amygdala—a subcortical cluster of nuclei in the medial temporal lobe critically implicated in the detection of emotional signals, particularly those associated with threat (Vuilleumier 2005; Domínguez‐Borràs and Vuilleumier 2022). Research in both humans (Vuilleumier et al. 2003; Vuilleumier and Driver 2007) and monkeys (Hadj‐Bouziane et al. 2012) further demonstrated that the amygdala not only responds to threat‐related cues such as facial or bodily expressions but also exerts feedback influences on sensory cortical areas, thereby enhancing perceptual processing and attentional orienting toward emotionally salient stimuli (see also Pessoa and Adolphs 2010). Accordingly, FFA activation to faces has been found to be modulated by both top‐down attention directed to faces and by emotional expressions of faces (Vuilleumier et al. 2001, 2003; Vuilleumier 2005).

Altogether, this body of work suggests that emotional stimuli may receive preferential processing through a rapid recruitment of limbic regions such as the amygdala, which may arise partially independently of voluntary goal‐directed control and boost their salience in the competition for selective attention (Vuilleumier et al. 2001, 2004). However, this apparent efficiency or automaticity of emotional reactivity does not preclude an influence of top‐down attentional gating (Schettino et al. 2012; Vuilleumier 2015), as fear processing or fear conditioning may also depend on the availability of executive resources in certain conditions (Pessoa, Kastner, and Ungerleider 2002; Bishop et al. 2007; Mitchell et al. 2007; Silvert et al. 2007; Pichon, de Gelder, and Grèzes 2012; Hur et al. 2016). Nor does it rule out modulatory influences from contextual factors, such as affective states (Pichon et al. 2015) or implicit priming (Pichon, Rieger, and Vuilleumier 2012) induced prior to emotional processing tasks. Nevertheless, it remains debated whether emotional processing in the amygdala is under the control of top‐down attentional mechanisms that regulate the flow of sensory inputs from cortical areas to the amygdala, and thus is suppressed when cortical responses are attenuated due to inattention, or whether emotional responding may instead occur to some extent independent of selective attention and cortical processing.

In support of independent emotion‐attention mechanisms, an early study by Vuilleumier et al. (2001) simultaneously manipulated spatial attention and emotion using a visual matching task. Participants were asked to judge whether a pair of task‐relevant images was similar or different, while another pair of task‐irrelevant images was presented at another location. On each trial, one pair of images displayed houses, while the other pair displayed either neutral or fearful faces. The results revealed greater amygdala activation in response to fearful compared to neutral faces, regardless of whether attention was directed toward them, despite a reduction of activity in the fusiform face‐responsive visual cortex when faces were task‐irrelevant and attention was directed to houses instead. In addition, fearful compared to neutral faces evoked greater activation in the face‐responsive visual cortex both when they were task‐relevant and when they were task‐irrelevant, supporting independent modulatory influences of fear expression and voluntary attention on visual areas.

In contrast, other studies have reported that emotional processing in both the visual cortex and amygdala is modulated by top‐down allocation of attentional resources. Pessoa, Kastner, and Ungerleider (2002) used a task where a single fearful or neutral face was presented centrally, together with two peripheral bars, while participants performed either a gender discrimination task on the face or an orientation discrimination task on the peripheral bars. Their results showed that directing attention away from the emotional face during the bar judgment task (Pessoa, McKenna, et al. 2002) or increasing the attentional difficulty of the bar orientation judgments (Pessoa et al. 2005) led to reduced activation in both the visual cortex and amygdala, as well as in other regions associated with emotional processing. These findings suggest that emotional perception is not privileged and independent of selective attention, but is contingent on the availability of processing resources, just as other sensory inputs.

To reconcile these views, alternative accounts were also put forward, whereby emotional processing does not follow a strict dichotomy between automatic and controlled mechanisms, but rather relies on dynamic interactions with attentional systems that are sensitive to task demands and contextual features. For example, the regulation of emotional responses by frontoparietal attention networks may depend on cognitive load and stimulus properties (see Framorando et al. 2020). Accordingly, bottom‐up activation of the amygdala would be evoked only when attention is weakly engaged on a stimulus requiring shallow processing, but suppressed when attention is highly focused on a cognitively demanding task. Another model by Diano et al. (2016, 2017b) proposed that frontoparietal networks might exert top‐down control on separate stages of emotion processing, with inhibitory influences acting on amygdala responses at late stages of integration with cortical inputs, while sparing an early bottom‐up activation mediated by subcortical pathways, notably involving the superior colliculus and the pulvinar.

Crucially, these models emphasize the temporal dynamics of emotion–attention interactions, whereby early stages of emotional processing may rely on fast subcortical pathways, while later stages are more strongly shaped by top‐down cortical control. Consistent with this view, intracranial and MEG studies have shown that the amygdala can respond to salient emotional stimuli within very short latencies, even in the absence of explicit attentional manipulation (Méndez‐Bértolo et al. 2016). However, there is still no consensus on the exact influence of attentional control on emotional processing in the amygdala, nor on the relative contribution of cortical and subcortical routes across different task contexts. Subcortical structures, such as the superior colliculus and the pulvinar, may play an important role in integrating sensory, attentional, and socio‐emotional signals.

Converging evidence from neurophysiology, neuroimaging, and lesion studies indicates that the superior colliculus contributes to the construction of saliency maps and can influence both cortical visual areas and limbic structures, including the amygdala (Basso and May 2017; Isa et al. 2021; White, Berg, et al. 2017; White, Kan, et al. 2017; Tamietto et al. 2012). Importantly, recent in vivo parcellation of the human superior colliculus has revealed distinct subdivisions with differential connectivity to cortical and limbic networks, supporting its role as a distributed hub rather than a simple reflexive relay (Diano et al. 2025). Such an organization suggests that emotional processing may rely on parallel and partially independent pathways, which can remain functional even when top‐down cortical control is reduced or altered. While these subcortical pathways are not always directly assessed in experimental paradigms, they provide a useful conceptual framework for understanding how emotional processing may remain preserved under conditions of altered attentional control by cortical networks.

In this context, a critical issue remains concerning the contribution of cortical attention‐control regions in shaping emotional responses in visual and limbic systems. In particular, frontal nodes of the dorsal attention network have been shown to exert top‐down influences on sensory processing during selective attention tasks (Corbetta et al. 2005; Shulman et al. 2003). The frontal eye field (FEF) is classically involved in oculomotor control, including the regulation of saccadic reaction times (Gerits et al. 2011). Beyond this well‐established motor function, extensive evidence indicates that the FEF is a key component of attentional networks, involved in the voluntary (goal‐directed) orientation of spatial attention and the top‐down modulation of visual processing based on task demands and behavioral goals (Corbetta and Shulman 2002; Corbetta et al. 2000; Kastner et al. 1999; Ruff et al. 2009). Functional imaging and lesion studies have shown that FEF activity can influence early sensory processing stages in the extrastriate visual cortex (Corbetta et al. 2005; Shulman et al. 2003), and neurophysiological research in non‐human primates has demonstrated that FEF microstimulation enhances neuronal responses in area V4, even in the absence of eye movements (Moore and Armstrong 2003). Together, these findings provide causal evidence for top‐down attentional modulation from frontal regions to distant visual areas, and suggest that the FEF acts through predictive signals that selectively enhance relevant sensory inputs while suppressing irrelevant ones (Buschman and Kastner 2015). Despite this extensive literature, no causal study has yet examined whether disrupting top‐down attentional mechanisms in the FEF alters emotional processing in both cortical and subcortical regions.

Building on this body of work, the present study examined how transient perturbation of the FEF may modulate top‐down attentional influences on neural responses to fearful faces in the fusiform face area (FFA) and the amygdala (AMG). Based on prior work showing that continuous theta‐burst stimulation over the human frontal eye field induces behavioral effects consistent with reduced oculomotor excitability (Nyffeler et al. 2006), we hypothesized that cTBS applied to the right FEF would at least partly disrupt the implementation of top‐down attentional control.

Specifically, we aimed at testing whether FEF perturbation would affect emotional processing similarly in visual cortical areas and in the amygdala. To this aim, participants performed the same face‐house matching task as used in Vuilleumier et al. (2001) while undergoing fMRI in two separate sessions, one following theta‐burst rTMS and one without rTMS, with the order of sessions counterbalanced across participants. Importantly, each participant was randomly assigned to either an experimental group who received rTMS stimulation over the right FEF or a control group who received rTMS stimulation over the vertex—which was not expected to produce any impairment in attentional control while eliciting similar sensory and auditory stimulation characteristics of active TMS (Jung et al. 2016).

Based on the literature described above, three distinct hypotheses could be formulated regarding how a perturbation of top‐down attentional mechanisms induced by cTBS—assumed to produce an *inhibitory‐like* perturbation of FEF function—might affect emotional processing.

First, if perturbing FEF function alters the processing of fearful faces in the FFA but not in the amygdala, this would support the view that emotional processing in the amygdala can operate relatively independently of top‐down selective attention and cortical inputs from face‐selective visual areas.

Second, if both FFA and amygdala responses are reduced following rTMS over the FEF, this would be consistent with models proposing that attentional resources are a necessary condition for emotional processing to emerge and drive amygdala reactivity.

Finally, a third, more nuanced hypothesis inspired by Diano et al. (2017a, 2017b) predicts that disrupting top‐down control via the FEF may not only impair selective attentional gating of face processing in the FFA, but also release inhibitory influences over emotionally salient signals conveyed through subcortical pathways, potentially resulting in preserved or enhanced amygdala responses.

Therefore, by combining rTMS‐induced perturbation of FEF function with fMRI during a visual attention task, the present study aimed to characterize the functional consequences of altered top‐down attentional control on emotional reactivity in both visual cortical regions and the amygdala.

## 2 Method

### 2.1 Participants

Forty healthy right‐handed participants (21 females; mean age = 25.0 years, range = 18–35), with no history of neurological or psychiatric disorders, were initially included in the study and randomly assigned to one of two groups: rTMS over the right frontal eye field (FEF group, *n* = 20) or rTMS over the vertex (VTX group, *n* = 20). All participants had normal or corrected‐to‐normal vision and were screened for contraindications to TMS and MRI.

All participants completed the fMRI protocol and were included in the neuroimaging analyses. Due to technical failures of the response device during scanning, behavioral data from two participants in the VTX group could not be analyzed. Accordingly, behavioral analyses were conducted on 20 participants in the FEF group and 18 participants in the VTX group.

Participants gave written informed consent prior to testing, and received financial compensation for their time.

### 2.2 General Procedure of the Experiment

**FIGURE 1:**
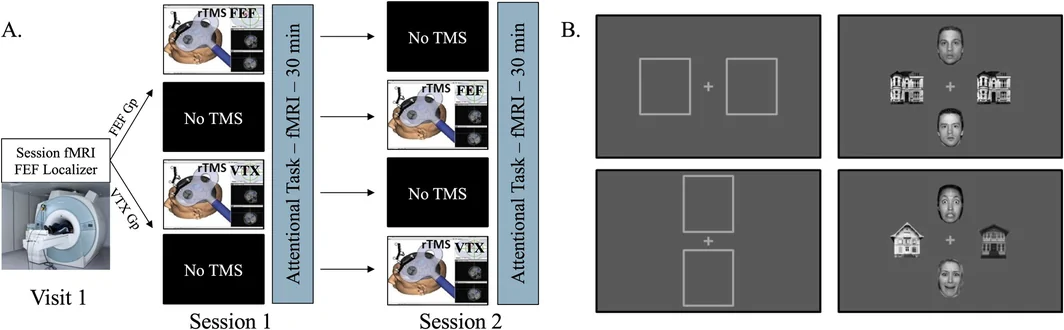
(A) General procedure for groups and sessions. (B) Attentional matching task experiment: Face‐House Matching task. Up: Attention directed to horizontal stimuli (houses, response = same), here shown with Neutral task‐Irrelevant faces (NI). Down: Attention directed to vertical stimuli (faces, response = different), shown with Fearful task‐Relevant faces (FR).

Participants were randomly assigned to a group who received rTMS stimulation (*inhibition‐like* protocol via cTBS) of either the right FEF (experimental group) or a control site (vertex, control group). Each participant completed two separate sessions of the same behavioral task on different days, preceded or not by rTMS stimulation. Session order was counterbalanced within each group, with half of the participants receiving rTMS in the first session and the other half in the second session (Figure 1A). The aim of the study was explained to participants; however, they were not informed that they could receive an active (FEF site) or placebo (vertex site) rTMS stimulation. During the first visit, all participants also performed a voluntary prosaccade task during fMRI to localize their frontal eye field (FEF) for subsequent (potential) rTMS targeting (Munoz and Everling 2004).

After completing the initial data acquisition required for neuronavigation, participants returned for two experimental sessions. One session (either the first or the second) involved performing a face‐house visual matching attention task (as in Vuilleumier et al. 2001) during fMRI alone (approximately 30 min). The other session involved the same fMRI task but was preceded by a session of neuronavigated rTMS targeting one randomly selected site (right FEF or vertex), depending on the participant's group assignment. The mean interval between the end of the rTMS session and the onset of the subsequent matching attention task was 8–10 min across participants, ensuring that the task was performed within the well‐documented > 30‐min window of cortical perturbation induced by this TMS protocol (Goldsworthy et al. 2012; Nyffeler et al. 2006; Korb et al. 2015). The study protocol was approved by the Ethics Committee of the University of Geneva and was conducted in accordance with the Helsinki Declaration.

### 2.3 Behavioral Task and Stimuli

We used the same face‐house matching task as described above (see Introduction) and used in previous studies (Bentley et al. 2003; Pichon et al. 2015; Vuilleumier et al. 2001, 2003; De Taeye et al. 2015; Hakamata et al. 2023). Participants were instructed to attend to task‐relevant images at the horizontal locations during a block of 64 trials, and then at the vertical positions for another block of 64 trials (128 trials total, order counterbalanced between subjects). The instruction was given before each sequence using a visual display made of two vertical or horizontal empty frames depicting the location of task‐relevant stimuli (Figure 1B). We insisted that participants maintained their gaze on the central fixation while attending to the task‐relevant stimulus pair in the horizontal or vertical locations. The goal of the task was to indicate whether images of the task‐relevant pair were identical or different from each other (i.e., attended pair), while ignoring the images of the task‐irrelevant pair (i.e., supposedly unattended). The pairs always involved either two houses or two faces, positioned vertically or horizontally around a central fixation cross. The position of faces and houses alternated between the vertical and the horizontal pairs in pseudo‐random order across trials in each task block. For both faces and houses, half of the pairs were composed of identical pictures, and the other half comprised different pictures. Orthogonally to these factors, facial stimuli depicted either fearful or neutral expressions (each for half of the face pairs, always with the same expression for the two faces). Faces and houses appeared equally often in vertical or horizontal pairs across all conditions.

This design resulted in four experimental conditions of interest within each session: faces with either fearful (F) or neutral (N) emotional expression, shown at either task relevant (R) or irrelevant (I) locations (i.e., FR, FI, NR, NI). For each behavioral measure, we computed average matching performance (accuracy, reaction times). For each participant, the full protocol resulted in a total of 8 conditions: rTMS before (FR, FI, NR, NI), and No‐rTMS before (FR, FI, NR, NI).

The visual stimuli included 32 neutral faces (16 males and 16 females) and the same 32 faces with fearful expressions from the NimStim Database (NimStim MacBrain set, www.macbrain.org), plus 32 different houses from our previous dataset (Pichon, de Gelder, and Grèzes 2012; Pichon, Rieger, and Vuilleumier 2012). All possible combinations of stimulus position, same/different identity for faces or houses, and facial expression were equally counterbalanced across conditions and presented in random order. We also controlled for face identities, so that for a given subject, the actors used to express fear always differed from the actors used for neutral expressions.

All images were presented on a dark grey background and projected through a mirror onto the head‐coil. The timing of presentation and spatial disposition of stimuli ensured reliable central fixation, since eye movements toward one of the peripheral positions would make the other less visible and thus decrease matching performance (see Duncan 1980; Wojciulik et al. 1998). Each trial began with a black central fixation cross lasting 1 s, followed by the four equiluminant black‐and‐white pictures displayed for 250 ms (visual angle 3° × 5°). Subjects responded by pressing one of two keys with their right hand. Inter‐trial intervals were pseudo‐randomly generated using a Poisson distribution with a mean of 3000 ms, lower and upper bounds of 2000 and 7000 ms. Each task block included 128 trials (duration 15 min).

Eye position was monitored online during scanning using an MR‐compatible eye‐tracking system to ensure compliance with central fixation instructions. However, due to limited spatial resolution for calibration, eye‐tracking data could not be reliably analyzed offline for several participants and were therefore not included in the present analyses. Please note that the task demands and stimulus position (orthogonal to experimental conditions) were specifically designed to effectively discourage saccades and make sustained central fixation the most efficient strategy for accurate performance (Vuilleumier et al. 2001; Wojciulik et al. 1998). Behavioral data were analyzed using STATISTICA software (version 14.1.0.8, TIBCO Software Inc.).

### 2.4 fMRI Data Acquisition

Functional images were obtained on a 3T Magnetom TIM Trio scanner (Siemens, Erlangen, Germany) equipped with a 32‐channel head coil. We acquired a gradient‐echo T2*‐weighted echo‐planar imaging (EPI) sequence with BOLD contrast, using a multiplexed protocol (Feinberg et al. 2010). Participants wore earplugs to attenuate scanner noise, and their head movement was restricted by a vacuum pillow. Each volume contained 36 axial slices acquired using a multiband EPI sequence with a repetition time of 2.6 s per whole‐brain volume (multiband factor = 4; TR per excitation = 650 ms, TE = 30 ms, flip angle = 54°, FOV = 192 mm, resolution = 64 × 64 pixels, voxel size of 3 × 3 × 3 mm, distance factor 30%, 0.9 mm thickness). Anatomical images were obtained using a T1‐weighted MPRAGE sequence (TR/TI/TE = 1900 ms/900 ms/2.27 ms, flip angle = 9°, FOV = 230 mm, PAT factor = 2, voxel dimensions: 1 mm isotropic, 256 × 256 × 192 voxels). For each subject, a first sequence of 815 functional volumes and two sequences of 560 volumes were recorded, for the FEF localizer and the two attentional task sessions, respectively (total of 1935 functional images).

### 2.5 TMS Stimulation Over the Right FEF or Vertex

TMS was applied either to the FEF due to its key role in top‐down attentional control (see above) or to the vertex (VTX) in separate groups, in alternation with a NoTMS session. VTX was chosen as an active control condition to match non‐specific sensory and auditory effects of TMS during the task across the two groups, while avoiding direct interference with attentional or emotional networks. While this accords with standard practice, this is not a fully inert control.

#### 2.5.1 FEF Localizer Session

FEF was located individually based on a preceding fMRI scanning session, conducted on a different day. Participants performed a series of eye movements in a voluntary pro‐saccade task, known to activate specifically the FEF (Munoz and Everling 2004). Visual stimuli consisted of a central fixation cross presented with two peripheral circles at 10° eccentricity. Participants were instructed to maintain their gaze on the cross until it was replaced by an arrow indicating the direction of the saccade (left or right), at which point they had to shift their gaze as quickly as possible towards one of the two peripheral circles. Simultaneously, a target number appeared in the peripheral circle on the left or right side, and participants had to report whether it contained a 6 or a 9 by pressing a button (yes/no; half of the subjects responded to 9, the other half to 6). One hundred and sixty trials were completed (approximately 10 min). This procedure was previously shown to elicit robust activation in the FEF (Guyader et al. 2010).

The fMRI data obtained during this task were then analyzed immediately after acquisition using BrainVoyager QX (Brain Innovation, The Netherlands) in order to define saccade‐related activity for each individual subject. Functional images were motion‐corrected and co‐registered to the individual T1 image (no spatial normalization was performed). Activation maps were calculated based on the arrow stimulus timing (saccade onset) convolved with a canonical HRF. Head motion parameters were included as regressors of no interest.

#### 2.5.2 Neuronavigation and Coil Placement

For each TMS session, the coil was placed either over the right FEF or the VTX with the help of a frameless neuronavigation system based on individual anatomical and functional MRI data. The system combines the neuronavigation module of BrainVoyager Qx software with the ultrasound CMS20 measuring system for navigation (Zebris GmbH, Tübingen, Germany). Target coordinates for TMS in the FEF were determined by defining from fMRI data the peak of the largest cluster of activation around the well‐known location of the FEF (junction of the middle frontal gyrus and the precentral gyrus). The average MNI coordinates at which rTMS of the right FEF was applied were 21.6 ± 5; −11 ± 10; 53.4 ± 5 over the whole group, in good correspondence with the previously reported location of the FEF (Paus 1996). The VTX target area was defined as the midpoint between the inion and the nasion in the antero‐posterior head axis and along the midline with the help of the neuronavigation system. The first phase of the biphasic currents flowing into the coil was oriented in an antero‐medial direction for stimulation of the FEF, perpendicularly to the central sulcus. Currents were oriented forward for stimulation of the VTX (Korb et al. 2015).

#### 2.5.3 Continuous Theta Burst Stimulation (cTBS) Protocol

Transcranial magnetic stimulation (TMS) was applied using a Magstim Rapid2 generator and a 70 mm figure‐of‐eight‐coil. In order to inhibit the attentional network, we chose to stimulate the right FEF with a continuous theta burst stimulation (cTBS) protocol, previously shown to induce robust and prolonged suppression of local cortical activity (Goldsworthy et al. 2012; Korb et al. 2015; Nyffeler et al. 2006). Theta burst stimulation (cTBS) consisted of 200 bursts of three TMS pulses each delivered at 30 Hz, with bursts delivered at 6 Hz. Thus, over a period of 33.3 s, a total of 600 pulses were administered at an intensity of 80% of the motor threshold (Nyffeler et al. 2006).

### 2.6 fMRI Data Analysis

Preprocessing and statistical analyses of the functional MRI data were conducted using SPM8 (Wellcome Department of Cognitive Neurology, London, UK) implemented in MATLAB (MathWorks Inc., Sherborn, MA, USA). Functional images were realigned to the first volume using a rigid‐body transformation to correct for head motion, and slice‐timing correction was applied to adjust for differences in slice acquisition timing. Volumes were then normalized to the Montreal Neurological Institute (MNI) standard EPI template, resampled to an isotropic voxel size of 2 × 2 × 2 mm, and spatially smoothed with an 8 mm full‐width at half‐maximum (FWHM) Gaussian kernel.

#### 2.6.1 First‐Level Analysis

For each participant, data were analyzed using the general linear model (GLM) for event‐related designs. For each session, four experimental conditions were modeled (FR, FI, NR, NI) by convolving the onset times of each event type with the canonical hemodynamic response function (HRF) and its temporal derivative to account for latency variations. Movement parameters derived from realignment were included as regressors of no interest. This resulted in eight regressors of interest (4 conditions × 2 HRF terms) per session, plus six motion regressors and an intercept term. Low‐frequency drifts were removed using a high‐pass temporal filter (cutoff = 128 s), as implemented in the first‐level GLM in SPM.

#### 2.6.2 Whole‐Brain Analysis

At the group level, individual t‐maps generated by contrasts between task conditions were entered into a second‐level random‐effects analysis using a flexible factorial design. Our model included one between‐subject factor (Group) and two within‐subject factors (Attention and Emotion). Statistical maps were thresholded at *p* < 0.001 (uncorrected) at the voxel level, with cluster‐level family‐wise error (FWE) correction at *p* < 0.05. Whole‐brain analyses were used to characterize task‐related activation patterns and to localize category‐selective regions involved in face and house processing. Due to our strong a priori hypothesis concerning brain sites affected by attention and emotion signals, as well as the limited statistical power of voxelwise interaction analyses with stringent correction for multiple comparisons, the critical rTMS‐related interaction effects were assessed within predefined regions of interest rather than at the whole‐brain level.

#### 2.6.3 Region of Interest (ROI) Analysis

All ROI analyses focused on a priori regions implicated in attentional and emotional processing: the frontal eye fields (FEF), fusiform face area (FFA), parahippocampal place area (PPA), and amygdala (AMG).

The FFA and PPA were localized using standard category‐selective contrasts (faces > houses and houses > faces), independent of emotional manipulations. Group‐level peak coordinates derived from these contrasts were used to define bilateral ROIs, consistent with prior work using the same paradigm (Vuilleumier et al. 2001; Pichon et al. 2015). The FEF ROI corresponded to the rTMS target and was defined based on group‐level coordinates obtained from a pro‐saccade localizer task prior to neuronavigation. Given the variability and limited whole‐brain statistical power for amygdala activations, bilateral amygdala ROIs were defined using spherical regions (8‐mm radius) centered on coordinates corresponding to the amygdala in standard MNI space (right: 30, −2, −30; left: −30, −4, −30), implemented using the MarsBaR toolbox.

ROI analyses were specifically designed to assess rTMS‐related and interaction effects within these regions, rather than to re‐test the effects used for regional identification, thereby limiting any potential circularity (Kriegeskorte et al. 2009).

## 3 Results

### 3.1 Behavioral

Behavioral analyses were conducted on 20 participants in the FEF group and 18 participants in the vertex (VTX) group (two participants from the VTX group were excluded due to technical failures of the response device). Statistical significance was set at *p* < 0.05. Effect sizes are reported as partial eta squared (*η* ^2^ *_p_*).

#### 3.1.1 Baseline Comparison (NoTMS Condition)

To verify that the two groups did not differ at baseline, reaction times in the NoTMS condition were compared between the FEF and VTX groups. A one‐way ANOVA revealed no significant group difference, *F*(1, 33) = 0.09, *p* = 0.98, *η* ^2^_*p*_ = 0.01, indicating equivalent baseline performance prior to rTMS. Accuracy showed the same pattern, with no significant group difference in the NoTMS condition, *F*(4, 33) = 0.38, *p* = 0.82, *η* ^2^ *_p_* = 0.04. These results indicate that the two groups were well matched at baseline prior to rTMS.

Following confirmation of equivalent baseline performance, behavioral effects of rTMS were examined separately within each group. A 2 × 2 × 2 (TMS [NoTMS/TMS] × Emotion [Fear/Neutral] × Attention to Face [Task‐relevant/Task‐irrelevant]) repeated‐measures ANOVA was performed on mean Reaction Times (RTs) and correct response rates for each TMS group.

#### 3.1.2 Reaction Times

##### 3.1.2.1 FEF Group

**FIGURE 2:**
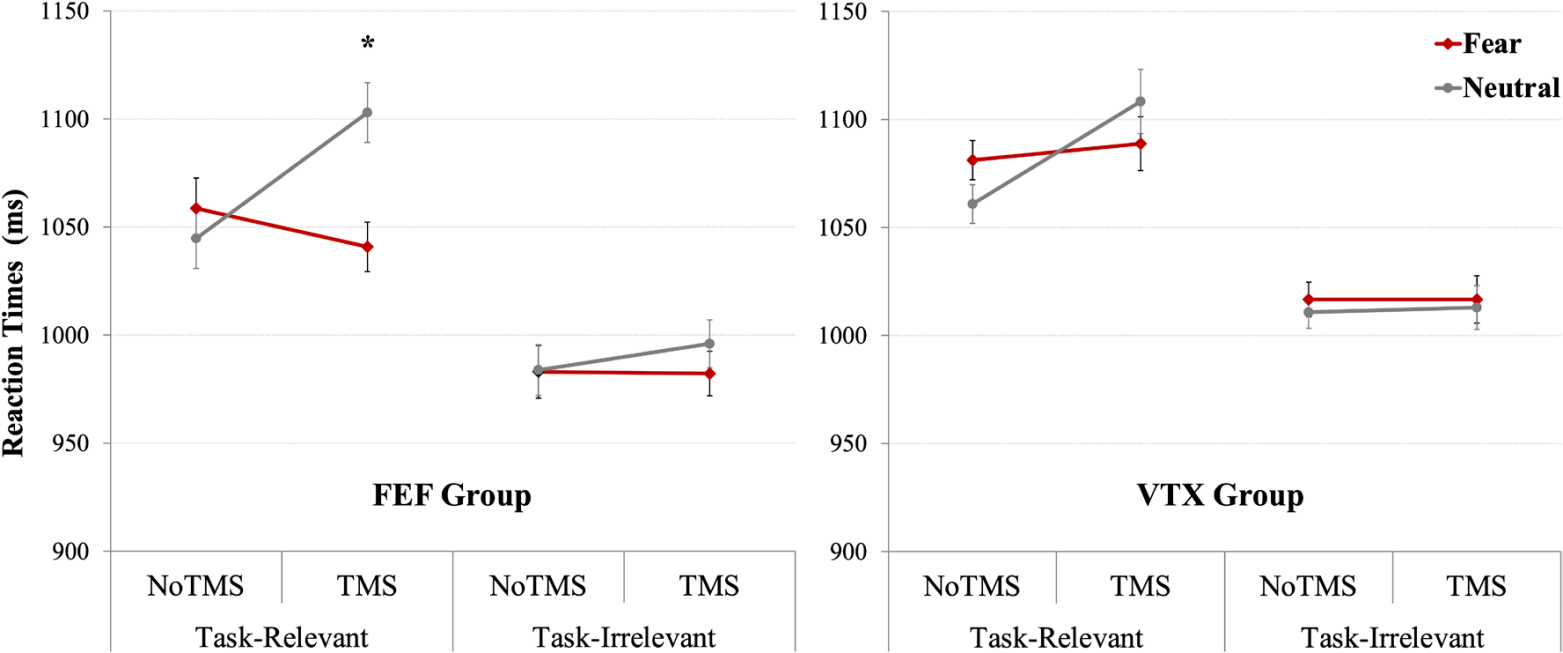
Behavioral reaction times (in ms, ±1 SEM) in the attentional task depending on TMS group (frontal eye field—FEF and vertex—VTX), session (NoTMS and TMS), and attention to fearful and neutral faces conditions (face task‐relevant and task‐irrelevant).

RTs showed a significant main effect of Attention, with slower responses to judge faces than houses (1062 ms vs. 986 ms; *F*(1, 19) = 18, *p* = 0.0004, *η* ^2^_*p*_ = 0.49). Importantly, there was a significant triple interaction between TMS, Emotion, and Attention (*F*(1, 19) = 5.7, *p* = 0.028, *η* ^2^ *_p_* = 0.23). Post hoc analyses (Figure 2) showed that in the TMS session, RTs differed between neutral and fearful faces in the task‐relevant condition (1103 vs. 1041 ms; *F*(1, 19) = 5.10, *p* = 0.036), whereas no such difference was observed in the task‐irrelevant condition (996 vs. 982 ms; *F*(1, 19) = 0.6, *p* = 0.436). No significant emotion effect was observed in the NoTMS session for either condition (*F*(1, 19) = 0.18, *p* = 0.677). No other main effects or interactions involving TMS reached significance in this group.

##### 3.1.2.2 Vertex (VTX) Group

RTs also showed a significant main effect of Attention, with slower responses for judging faces than houses (1084 ms vs. 1014 ms; *F*(1, 17) = 9.5, *p* = 0.007, *η* ^2^ *_p_* = 0.36). However, no interaction involving TMS, Emotion, or Attention reached statistical significance in this group.

To directly test for a differential emotional modulation depending on stimulation site, we computed for each participant a TMS‐induced change index reflecting the Neutral–Fear contrast in the task‐relevant face condition (ΔEmotionEffect = [(Neutral–Fear)_TMS – (Neutral–Fear)_NoTMS]). This effect was twice as large in the FEF compared to the vertex group but failed to reach statistical significance (FEF: *M* = 76 ms; VTX: *M* = 39.8 ms; *p* = 0.493).

#### 3.1.3 Control Analysis for Inter‐Individual Differences in Reaction Times

To assess whether the observed behavioral effects were robust to inter‐individual differences in overall reaction times, data were additionally normalized within subjects using *z*‐score transformation based on the eight condition‐wise mean RTs (TMS × Emotion × Attention), and re‐analyzed using the same ANOVA models.

In the FEF group, this analysis confirmed the original pattern of results, with a significant main effect of Attention (*F*(1, 19) = 17.7, *p* = 0.0005, *η* ^2^_*p*_ = 0.48) and a significant TMS × Emotion × Attention interaction (*F*(1, 19) = 5.22, *p* = 0.034, *η* ^2^ *_p_* = 0.22).

In contrast, in the vertex (VTX) group, the *z*‐scored RT analysis revealed a significant main effect of Attention (*F*(1, 17) = 8.41, *p* = 0.010, *η* ^2^ *_p_* = 0.33), but no significant interaction involving TMS, Emotion, or Attention (TMS × Emotion × Attention: *F*(1, 17) = 1.23, *p* = 0.283). These results confirm that differences between groups are not related to different inter‐individual variability within groups.

#### 3.1.4 Response Accuracy

##### 3.1.4.1 FEF Group

A significant main effect of Attention was observed, with higher accuracy when matching houses (faces task‐irrelevant) than when matching faces (houses task‐irrelevant) (88.8% vs. 75.8%; *F*(1, 19) = 65.1, *p* < 0.0001, *η* ^2^ *_p_* = 0.77). No main effect of Emotion and no interaction involving TMS reached significance in this group.

##### 3.1.4.2 Vertex (VTX) Group

Accuracy results showed the same pattern, with a robust main effect of Attention (86.6% vs. 72.6%; *F*(1, 17) = 51.8, *p* < 0.0001, *η* ^2^_*p*_ = 0.75). Again, accuracy was not modulated by face expression, TMS, or their interaction.

Overall, response accuracy confirmed that matching houses was easier than matching faces, independently of emotional expression and TMS condition in both groups.

### 3.2 fMRI

#### 3.2.1 Whole Brain

To verify that our visual task produced activation patterns similar to previous studies using the same paradigm (Wojciulik et al. 1998; Vuilleumier et al. 2001, 2003), we ran a whole‐brain analysis of fMRI data at baseline (without any TMS effect) using a standard flexible factorial design in SPM, with 2 attention conditions (face at task‐relevant or task‐irrelevant position) and two emotion conditions (face with fearful or neutral expression). This analysis ensured that we could identify key ROIs according to our a priori hypotheses and then examine TMS effects on independently defined regions. Thus, SPM results are not reported as inferential results but serve to define neural targets where we could test our predictions concerning modulations of attention and emotion processing in the visual cortex by TMS. Direct contrasts between task conditions showed expected effects of attention in the visual cortex. First, comparing *Task‐Relevant > Task‐Irrelevant face* conditions revealed significant bilateral activation in the fusiform cortex, with clusters corresponding to the right (*x* = 44, *y* = −46, *z* = −24) and left FFA (*x* = −42, *y* = −48, *z* = −22), both significant at *p* < 0.05 FWE corrected. Conversely, the reverse contrast *Task‐Irrelevant > Task‐Relevant face conditions*, corresponding to conditions in which attention was directed toward houses, showed bilateral activation in the parahippocampal place area (PPA), with peaks at (30, −42, −12) on the right and (−26, −48, −14) on the left (*p* < 0.05 FWE), consistent with well‐established attentional modulation of scene‐selective cortex.

This latter contrast also highlighted increased activation in components of the dorsal attention network, including areas overlapping with the right and left frontal eye field (FEF), observed at uncorrected voxel‐level thresholds (*p* < 0.001 and *p* < 0.002, respectively), with peaks at (24, 6, 52) and (−28, 12, 50); as well as bilateral intraparietal sulcus (IPS) regions (right: *p* < 0.05 FWE; left: *p* < 0.06 FWE). These activations are reported descriptively to illustrate engagement of attentional control networks during the task.

Regarding emotional processing, the main effect of Emotion (*Fear* > *Neutral*), collapsed across attention conditions revealed bilateral amygdala (AMG) responses. These effects were observed at uncorrected thresholds, with a peak in the right amygdala (30, −2, −30, *p* < 0.05 unc.) and a similar trend in the left amygdala (−30, −4, −30, *p* < 0.06 unc.). Although these activations did not survive correction for multiple comparisons at the whole‐brain level, they are consistent with the expected sensitivity of the amygdala to emotionally salient stimuli (Vuilleumier et al. 2001) and motivated subsequent hypothesis‐driven ROI analyses.

#### 3.2.2 ROI Analyses

We next examined critical effects of TMS over the FEF or the VTX on functional neural responses in key brain regions engaged during the task. To improve readability, the main effects and critical interactions observed across ROIs for each group are summarized in Table 1, while detailed statistical results are described in the next sections below.

**TABLE 1:** Summary of attentional, emotional, and rTMS‐related effects across regions of interest and groups.

ROI	Group	Attention	Emotion	TMS	TMS × attention	TMS × emotion	TMS × emotion × attention	Key finding
FEF	FEF	✓	—	✓	—	—	—	Bilateral reduction of FEF activity after rTMS
	VTX	✓	—	—	—	—	—	No TMS‐related effect
FFA	FEF	✓	*t*	—	—	—	✓ (+ Hem.)	Selective attenuation for neutral faces after FEF rTMS
	VTX	*t*	—	—	—	—	—	No TMS‐related modulation
PPA	FEF	✓ (+ Hem.)	—	—	—	—	—	Strong attentional modulation only
	VTX	✓	—	—	—	—	—	Replication of attentional effect
AMG	FEF	✓	*t*	—	—	✓ (+ Hem.)	—	Enhanced fear response after FEF rTMS (left)
	VTX	✓	—	—	—	—	—	No TMS‐related modulation

*Note:* ✓ indicates a significant effect (*p* < 0.05); *t* indicates a trend‐level effect (0.05 < *p* < 0.10); — indicates no significant effect. Only effects relevant to a priori hypotheses are reported. Interactions involving the Hemisphere factor are indicated (+Hem.) when reported.

All statistical analyses were conducted on pre‐defined ROIs to examine condition‐specific modulations within independently defined regions of interest, rather than to re‐establish the main effects used for regional identification. This approach was adopted to avoid circularity and to specifically assess the impact of rTMS and its interactions with attention and emotion processing within these regions. Percent signal changes relative to each participant's global mean signal were computed for our main ROIs (FFA, AMG, FEF, PPA), separately for the FEF and VTX TMS groups. Resulting values were submitted to 2 × 2 × 2 × 2 repeated‐measures ANOVAs with Hemisphere (left vs. right), Emotion (fearful vs. neutral), Attention to faces (face task‐relevant vs. task‐irrelevant), and TMS condition (TMS vs. NoTMS session) as within‐subject factors. In these analyses, all factorial contrasts were tested but we focus only on those relevant to our a priori hypotheses or statistically reliable effects. Additional contrasts did not reveal robust or interpretable effects and are therefore not discussed in detail.

#### 3.2.3 Target Group With TMS Over FEF

##### 3.2.3.1 Frontal Eye Field—FEF ROI—FEF Group

We observed several significant effects in this region, defined from whole brain results above but partly overlapping with the FEF target in our localizer scan prior to TMS. First, there was a main effect of *Hemisphere*, *F*(1, 19) = 6.57, *p* = 0.019, *η* ^2^ *_p_* = 0.257, indicating greater overall BOLD activity during the task in the left compared to the right (inhibited) FEF. A significant main effect of *Attention* was also found, *F*(1, 19) = 13.88, *p* = 0.001, *η* ^2^ *_p_* = 0.422, with lower FEF activation when faces were task‐relevant compared to when faces were task‐irrelevant (i.e., when houses were task‐relevant), consistent with whole‐brain results above.

More importantly, a main effect of *TMS* was observed, *F*(1, 19) = 8.17, *p* = 0.010, *η* ^2^_*p*_ = 0.30, with decreased activation on both sides after TMS stimulation over the right FEF target, as compared to the NoTMS session. This effect was bilateral (no interaction of TMS × Hemisphere, *F*(1, 19) = 1.07, *p* = 0.31). No other main effects or interactions reached statistical significance. These data indirectly confirm the impact of FEF inhibition by TMS, extending bilaterally in both hemispheres.

##### 3.2.3.2 Fusiform Face Area—FFA ROI—FEF Group

A main effect of Hemisphere was observed, *F*(1, 19) = 10.46, *p* = 0.004, *η* ^2^ *_p_* = 0.355, with greater activation in the right than left FFA across conditions, consistent with a right hemispheric dominance for face processing. As expected by design, FFA activity was also modulated by attentional conditions, with stronger responses when faces were task‐relevant than when houses were task‐relevant, *F*(1, 19) = 10.71, *p* = 0.004, *η* ^2^_*p*_ = 0.361.

**FIGURE 3:**
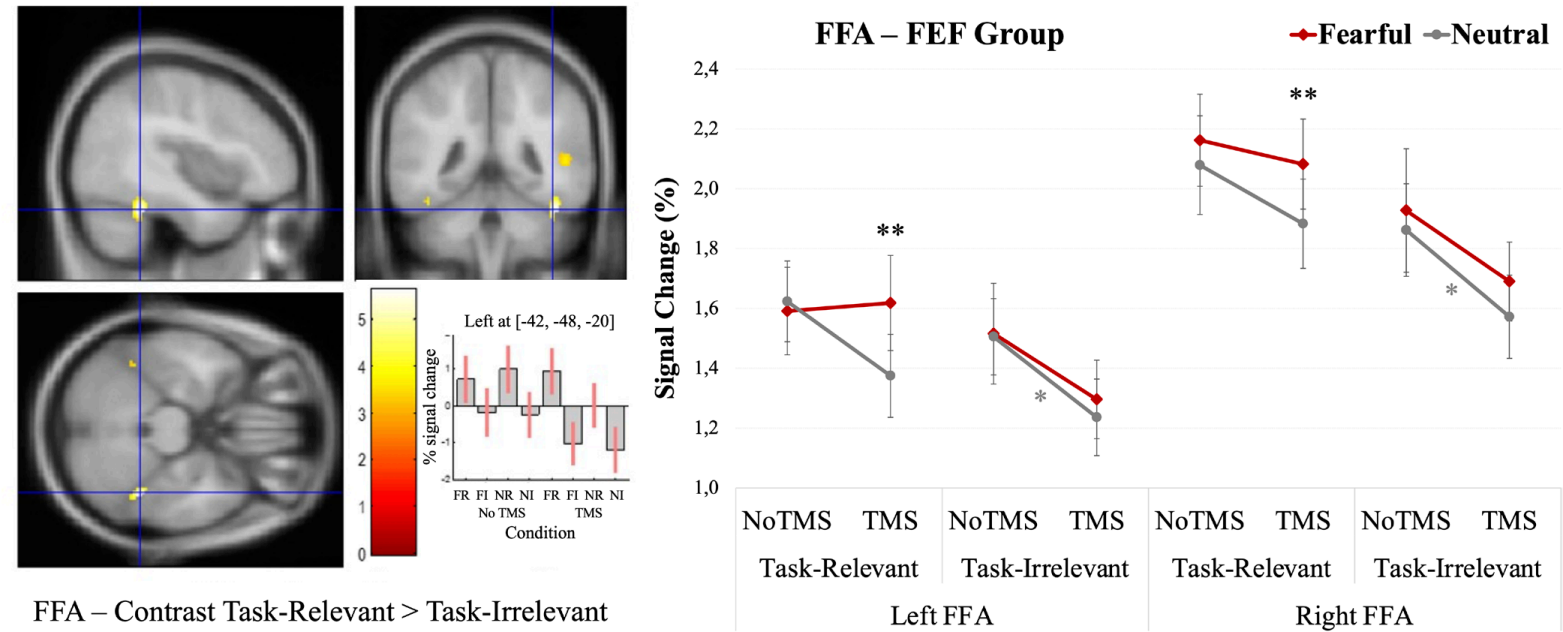
Left panel: FFA ROI defined by whole‐brain analysis at baseline across both groups. Right panel: Mean percentage signal change in the fusiform face area (FFA) in the FEF‐TMS group for fearful and neutral faces, in each attention condition (task‐relevant vs. task‐irrelevant faces), hemisphere (left vs. right), and TMS session (TMS vs. NoTMS). Error bars represent ±1 standard error of the mean (SEM). Significance levels: *p < 0.05, **p < 0.005. FFA regions of interest were defined using functional contrasts (faces > houses).

Critically, we found a significant four‐way interaction between TMS, Hemisphere, Attention, and Emotion, with a large effect size, *F*(1, 19) = 5.36, *p* = 0.032, *η* ^2^ *_p_* = 0.220 (Figure 3). To clarify the source of this interaction, planned simple‐effects analyses were conducted within each level of Attention and Emotion, testing the effect of TMS separately for neutral and fearful faces, and for task‐relevant and task‐irrelevant conditions, in each hemisphere. These analyses revealed a significant decrease in FFA responses following TMS specifically for neutral faces in the task‐irrelevant condition, both in the left (*F*(1, 19) = 4.42, *p* = 0.049) and right hemisphere (*F*(1, 19) = 4.76, *p* = 0.042). No significant TMS‐related modulation was observed for fearful faces in the task‐irrelevant condition, either in the left (*F*(1, 19) = 1.62, *p* = 0.22) or in the right FFA (*F*(1, 19) = 2.39, *p* = 0.14). In the task‐relevant condition, left FFA activation for neutral faces also tended to be lower after TMS than in the NoTMS condition (*F*(1, 19) = 3.71, *p* = 0.069), but again there was no significant attenuation for fearful faces (*F*(1, 19) = 0.03, *p* = 0.855).

Moreover, comparing responses to task‐relevant faces after TMS showed significantly greater activation to fearful relative to neutral faces in both the left FFA, *F*(1, 19) = 14.14, *p* = 0.001, and right FFA, *F*(1, 19) = 6.96, *p* = 0.016; while there was no significant difference between fearful and neutral expressions in the task‐irrelevant face condition, neither in the left FFA, *F*(1, 19) = 0.76, *p* = 0.39, nor in the right FFA, *F*(1, 19) = 2.15, *p* = 0.16. Taken together, these data point to an attenuation of FFA reactivity by FEF TMS predominating when faces were less salient due to emotional expression (neutral compared to fearful) or attention demands (task irrelevant vs. task relevant).

Finally, a possible hemispheric asymmetry was suggested by a trend‐level Attention × Hemisphere interaction, *F*(1, 19) = 3.68, *p* = 0.070, *η* ^2^ *_p_* = 0.162; however, this effect did not reach statistical significance and is therefore reported descriptively. Additionally, there was a trend toward significance for the main effect of *Emotion*, *F*(1, 19) = 3.17, *p* = 0.091, *η* ^2^ *_p_* = 0.143, suggesting a tendency for greater activation to fearful than neutral faces overall, consistent with prior reports (Vuilleumier et al. 2001; Hadj‐Bouziane et al. 2012). There was no Emotion *×* Attention interaction, *F*(1, 19) = 0.56, *p* = 0.47, and no *significant* Emotion *× Hemisphere interaction, F*(1, 19) = 2.75, *p* = 0.11. No other main effects or interactions reached statistical significance.

##### 3.2.3.3 Parahippocampal Place Area—PPA ROI—FEF Group

PPA activity showed robust modulation by attentional condition, *F*(1, 19) = 137.36, *p* < 0.001, *η* ^2^ *_p_* = 0.878, with greater activation when faces were task‐irrelevant (and houses at to‐be‐attended locations) as compared to when faces were task‐relevant (and houses at to‐be‐ignored locations). A main effect of Hemisphere was also observed, *F*(1, 19) = 14.99, *p* = 0.001, *η* ^2^ *_p_* = 0.441, with greater activation in the left than the right PPA, and a significant Hemisphere × Attention interaction, *F*(1, 19) = 4.84, *p* = 0.040, *η* ^2^ *_p_* = 0.203, reflecting stronger attentional modulation in the left than the right PPA. No effects or interactions involving Emotion or TMS were detected.

##### 3.2.3.4 Amygdala—AMG ROI—FEF Group

A main effect of Attention was observed, with greater amygdala responses to task‐relevant faces compared to task‐relevant houses, *F*(1, 19) = 5.60, *p* = 0.029, *η* ^2^_*p*_ = 0.230. There was also a trend toward a main effect of Emotion, *F*(1, 19) = 4.10, *p* = 0.057, *η* ^2^ *_p_* = 0.178, suggesting a tendency for greater amygdala activation to fearful compared to neutral faces. Although this effect did not reach the conventional significance threshold, it is consistent with the expected sensitivity of the amygdala to emotional salience and is considered informative in the context of our study's a priori hypotheses.

**FIGURE 4:**
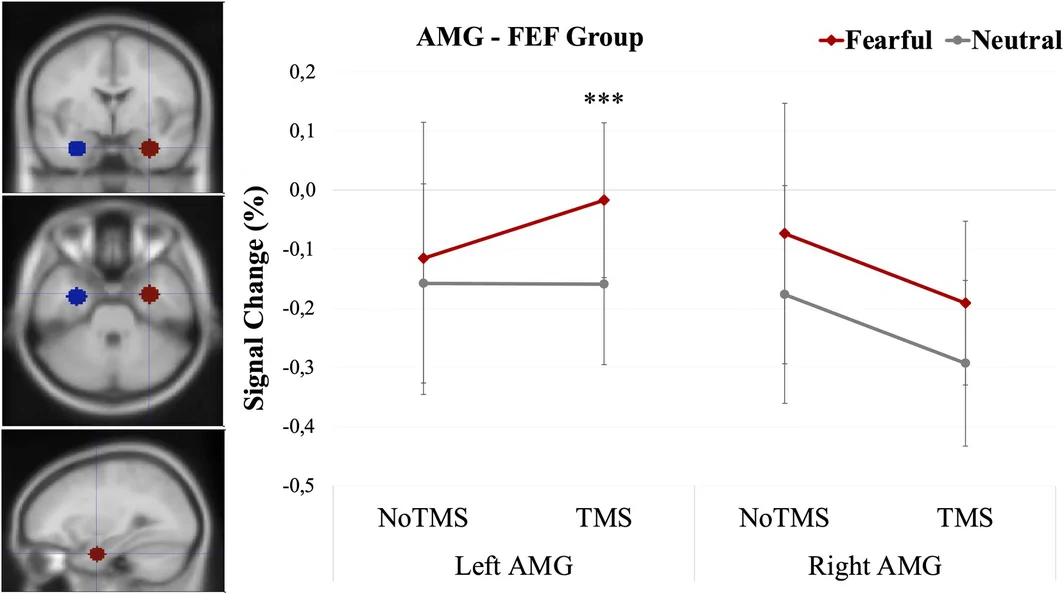
Left panel: Amygdala ROIs defined by using spheres centered on standard coordinates across both groups. Right panel: Mean percentage signal change in the amygdala for fearful and neutral faces, separately for each hemisphere (left vs. right) and TMS condition (TMS vs. NoTMS). Error bars represent ±1 standard error of the mean (SEM). Asterisks indicate significance levels: ***p < 0.001. Amygdala regions of interest were defined as bilateral spherical ROIs (8‐mm radius) centered on standard MNI coordinates.

No significant Emotion × Attention interaction was found, *F*(1, 19) = 0.75, *p* = 0.397. However, **a** significant three‐way interaction emerged between Hemisphere, TMS, and Emotion, *F*(1, 19) = 5.35, *p* = 0.032, *η* ^2^ *_p_* = 0.220 (see Figure 4). Post hoc comparisons revealed that this interaction was driven by significantly greater activation of the left amygdala to fearful than neutral faces after TMS, *F*(1, 19) = 16.51, *p* < 0.001. In the right AMG, the same contrast revealed a trend in the same direction, *F*(1, 19) = 4.21, *p* = 0.054; but this effect did not reach statistical significance.

No other comparisons reached statistical significance.

#### 3.2.4 Control Group With TMS Over Vertex

##### 3.2.4.1 FEF ROI—VTX Group

In this group, a significant main effect of Attention was again observed, *F*(1, 19) = 10.08, *p* = 0.005, *η* ^2^ *_p_* = 0.346, with greater activation for task‐irrelevant than task‐relevant faces, in keeping with the pattern observed in the FEF target group. Unlike the latter group, however, there was no significant effect of TMS on FEF activation, *F*(1, 19) = 2.03, *p* = 0.17, nor any hemispheric asymmetry, *F*(1, 19) = 0.56, *p* = 0.46.

##### 3.2.4.2 FFA ROI—VTX Group

A main effect of Hemisphere was found in the FFA, with greater activation in the right compared to the left side, *F*(1, 19) = 14.53, *p* = 0.001, *η* ^2^ *_p_* = 0.433. In addition, FFA responses tended to be higher when faces were task‐relevant than task‐irrelevant, *F*(1, 19) = 3.43, *p* = 0.080, *η* ^2^ *_p_* = 0.153. No other main effects or interactions reached statistical significance.

##### 3.2.4.3 PPA ROI—VTX Group

A strong modulation by attentional condition was observed in the PPA, with greater activation during house matching on trials with task‐irrelevant faces than during face matching, *F*(1, 19) = 71.29, *p* < 0.001, *η* ^2^ *_p_* = 0.790. No other main effects or interactions reached significance.

##### 3.2.4.4 AMG ROI—VTX Group

In this group, amygdala activation showed no main effect of Emotion, *F*(1, 19) = 0.44, *p* = 0.52, but a significant Hemisphere × Emotion interaction, *F*(1, 19) = 6.16, *p* = 0.023, *η* ^2^ *_p_* = 0.245. Although post hoc tests did not yield significant comparisons, activation patterns suggested that hemispheric differences were mainly driven by fearful faces, with relatively greater activation in the left amygdala (see Figure S1). In addition, a significant three‐way interaction between Hemisphere, Emotion, and Attention was found, *F*(1, 19) = 10.04, *p* = 0.005, *η* ^2^ *_p_* = 0.346, reflecting that those enhanced responses of the left AMG to fearful faces depended on both emotion and attention. Post hoc comparisons showed a trend for greater activation in the left compared to the right AMG for fearful task‐relevant faces, *F*(1, 19) = 3.98, *p* = 0.060, while there was no asymmetry for task‐irrelevant faces, *F*(1, 19) = 0.38, *p* = 0.54. Importantly, no effects or interactions involving TMS were found.

## 4 Discussion

Whole‐brain fMRI analyses confirmed category‐selective activations during our visual paradigm consistent with previous studies (Vuilleumier et al. 2001, 2003; Pichon et al. 2015). Greater responses in bilateral fusiform regions for task‐relevant faces relative to houses, and in bilateral parahippocampal regions for task‐relevant houses relative to faces, support a reliable engagement of ventral visual pathways during the task and their modulation by top‐down endogenous attention (Wojciulik et al. 1998). Amygdala responses to emotional face expressions (fear > neutral) were observed, but were relatively weak and heterogeneous across conditions, likely reflecting habituation effects related to repeated stimulus exposure across multiple blocks and sessions inherent to the present design (e.g., Breiter et al. 1996; Lieberman 2010). In addition, FEF activity also showed robust task‐related engagement in keeping with a concomitant implication of cortical attention circuits (Corbetta and Shulman 2002). Moreover, the latter was clearly modulated by rTMS in a group‐specific and task‐selective manner, providing direct evidence for an effective impact of stimulation on these circuits that could in turn presumably affect top‐down attentional control on cortical and subcortical emotional processing.

### 4.1 Effects of Right FEF Perturbation (FEF Group)

Behavioral results indicate that our visual matching task may be easier for houses than faces, with faster and more accurate responses when judging pairs of houses than pairs of faces at task‐relevant locations. As consistently reported with this paradigm, this baseline difference was expected and not interpreted per se, nor does it affect the (orthogonal) interaction‐based conclusions on TMS effects as drawn from the present factorial design.

More importantly, transient perturbation of the right FEF induced by cTBS (rTMS) led to significantly longer response times for neutral faces compared to fearful faces, only in the condition where faces were task‐relevant. This suggests that perturbation of the right FEF could disrupt selective attention processes necessary to focus on the task‐relevant neutral faces, with no observable effect on matching judgments for fearful faces or houses, possibly due to greater perceptual saliency and/or distinctiveness of the latter (Moore and Armstrong 2003; Vuilleumier et al. 2001). Although subtle repetition or time‐on‐task effects across blocks cannot be fully excluded, the counterbalanced session order, trial‐wise randomization of conditions, and the site‐ and condition‐specific nature of the behavioral modulation strongly argue against a nonspecific fatigue, learning, or task familiarity account. Importantly, this behavioral pattern remained significant even after within‐subject normalization of reaction times, indicating that the observed effect cannot be attributed to inter‐individual differences in overall response speed.

In parallel, fMRI analyses of pre‐defined ROIs yielded converging evidence at the neural level. FEF activation was generally higher when faces were task‐irrelevant—that is, when participants matched houses, independently of emotion expression or stimulation conditions. Notably, a significant and bilateral reduction in FEF activation was observed after TMS to this region (unlike TMS to the VTX), across all conditions, consistent with an *inhibitory‐like* modulation of FEF activity induced by cTBS, as reported in previous studies (Goldsworthy et al. 2012; Korb et al. 2015; Nyffeler et al. 2006).

As part of the dorsal frontoparietal network, the FEF plays a key role in spatial attention and influences the visual cortex activity via predictive top‐down signals, enhancing relevant inputs or suppressing irrelevant ones (Corbetta et al. 2000; Moore and Armstrong 2003). Although the frontal eye field is also classically involved in oculomotor control, its role in top‐down attentional modulation of visual processing is well established, even in the absence of overt eye movements. In the present study, the observed effects were condition‐specific and expressed in category‐selective visual and limbic regions; therefore they are more consistent with attentional rather than purely oculomotor effects on cortical and subcortical activations. Accordingly, FEF engagement during the task accords with its established role in modulating perceptual processing and parallels the behavioral advantage observed for house matching relative to face matching at baseline.

More critically, our data show that a reduction in right FEF activity after rTMS was associated with reliable and region‐specific changes in distant cortical and subcortical regions, including the fusiform cortex and the amygdala. Such distributed effects are consistent with previous TMS studies demonstrating that perturbation of the FEF can influence activity in remote visual areas through network‐level interactions (Ruff and Driver 2006). Together, these findings support a modulatory role of the FEF within distributed attention–emotion networks and argue for an interpretation in terms of circuit‐level interactions rather than a single, direct causal pathway.

In the FFA, we observed a strong attentional modulation, with significantly greater activation when faces were task‐relevant. In addition, a weak trend toward increased activation for fearful compared to neutral faces was observed, suggesting a secondary and more limited influence of emotional content alongside the dominant top‐down effects of attention on this cortical area. Together, these findings are consistent with a dual top‐down modulation of FFA activity, in which attentional demands exert a primary influence, while emotional signals contribute separately but more weakly (Vuilleumier et al. 2003; Vuilleumier 2005). However, a significant four‐way interaction involving Hemisphere, TMS, Attention, and Emotion was evidenced. Specifically, FFA activity was markedly reduced in response to neutral faces after rTMS over the right FEF, across conditions and hemispheres—but with significantly greater impact in the task‐irrelevant condition and in the left hemisphere. In contrast, following right FEF perturbation induced by cTBS, the left (and also right) FFA exhibited a relatively preserved—or even amplified—response to attentively processed (task‐relevant) fearful compared to neutral faces, seemingly counteracting or “resisting” the attenuation of cortical reactivity induced by TMS. In other words, transient alteration of FEF function following rTMS was associated with distant changes in cortical visual responses that not only slowed behavioral responses to (task‐relevant) neutral faces without affecting those to fearful faces, but also led to reduced neural activity in the FFA for most conditions except task‐relevant fearful faces—which showed no such attenuation and thus remained relatively “immune” to the rTMS‐induced attentional perturbation. One possible reason why task‐relevant faces did not show stronger rTMS effects is that attentional prioritization was already maximally engaged in this condition, leaving limited dynamic range for additional modulation. Importantly, this does not imply that emotional processing depends on attentional resources, but rather that perturbation effects are more readily revealed when attentional gating is required to resolve weaker or less salient signals, such as neutral or task‐irrelevant faces.

The apparent lack of detectable rTMS effects on FFA responses to fearful faces, unlike the rTMS effects seen for neutral faces, supports the hypothesis that emotional salience‐driven processing might be at least partly resilient to the disruption of top‐down attentional control mediated by the dorsal attention network areas, such as the FEF (Vuilleumier 2005; Domínguez‐Borràs and Vuilleumier 2022). The current findings converge with neuropsychological studies indicating that enhanced sensory responses to emotionally salient information (e.g., faces—Vuilleumier and Armony 2001; Lucas and Vuilleumier 2008; or voices—Grandjean et al. 2008) can persist in the presence of attentional deficits due to brain damage, such as spatial neglect after parietal or frontal stroke. In turn, these findings add further support to the idea of a dissociation between non‐voluntary emotional processing and goal‐relevant attentional modulation acting through partly separate neural mechanisms. However, the significant four‐way interaction indicates that the combined effects of emotional salience and attentional relevance may vary depending on stimulus and/or task conditions (see Schettino et al. 2012; Vuilleumier 2015). In our study, FFA responses to fearful faces therefore appeared relatively spared from the inhibitory effects of TMS, particularly when faces were task‐relevant. In contrast, neutral faces appear more sensitive to the inhibitory effects of right FEF inhibition, eliciting weaker activity under conditions of low attentional engagement, particularly in the left FFA (perhaps less face‐selective than the right FFA).

On the other hand, the PPA showed the expected attentional effects with preferential responses to task‐relevant houses, as predicted, but no differential modulation by TMS or face expression. This indicates stable visual and attentional processing in this region despite FEF inhibition, and aligns with the notion that emotional and attentional influences observed in the FFA were specific to face processing, not due to more global effects on attention resources, vigilance, or task‐set control.

In contrast, the amygdala (AMG), which responded more to task‐relevant than task‐irrelevant faces, also showed generally higher responses to fearful compared to neutral faces. Notably, in parallel to the FFA, this emotional effect was preserved or even amplified (in the left hemisphere) after rTMS over the FEF (compared to the NoTMS condition) – regardless of whether attention was directed to faces or to houses. This was evidenced by a significant three‐way interaction of Emotion, Hemisphere, and TMS, but no interaction involving Attention. Overall, these findings converge with those for the FFA above, indicating that emotional processing in the amygdala may persist despite impaired attentional control caused by TMS to the FEF, and could thus still allow for feedback modulation of the fusiform cortex. Moreover, the current pattern suggests that under standard conditions, emotional responses in the amygdala might partly be under inhibitory control from attentional systems, but released from such control (particularly on the left side) when rTMS disrupts the FEF. In other words, while an alteration of attentional mechanisms induced by rTMS could impair task‐dependent modulation of cortical activation in the FFA, it might also result in subcortical emotional disinhibition secondary to attenuated top‐down control and thus allow for enhanced (or relatively spared) amygdala reactivity.

This distinctive effect on cortical and subcortical areas would accord with the recent model proposed by Diano et al. (2017b), assuming a two‐stage organization of emotional processing. In this view, an initial rapid and “automatic” response is mediated by subcortical pathways (notably the amygdala), followed by a slower and later response in cortical structures (FFA) that is sensitive to attentional modulation (see also Vuilleumier 2005). In our study, the increased left amygdala activation to fearful faces observed after right FEF inhibition might thus reflect a release of early emotion responses from inhibitory attentional control, in line with the hypotheses proposed by Diano et al. (2017a).

Interestingly, results from time‐resolved imaging with magnetoencephalography (MEG) also support a temporally differentiated activation pattern in the amygdala in response to threatening faces. Luo et al. (2007) showed that this structure may activate very early (less than 50 ms after stimulus onset), preceding discriminative responses in the visual cortex, but exhibits another later component (around 300 ms) that may be more sensitive to the influence of top‐down attention exerted by the frontoparietal network (Luo et al. 2010). In the same vein, Liu et al. (2010, 2012) observed an initial amygdala response between 40 and 140 ms, which appeared relatively independent of attention, followed by a later modulation from 280 to 410 ms associated with attentional allocation. Similar patterns were observed with intracranial recordings in the human amygdala (Pourtois et al. 2010). These electrophysiological data thus broadly accord with the hypothesis of a two‐stage model of emotional processing as postulated by Diano et al. (2016, 2017b) and suggested here. Collectively, these results highlight a dynamic interplay between attentional and emotional networks. Although fMRI does not provide direct temporal information, the present interpretation is grounded in the consistency between the observed pattern of neural modulation and converging evidence from prior work using time‐resolved methodologies. Accordingly, the current conclusions pointing to rapid or attention‐independent emotion processing should be considered within this integrative, cross‐methodological framework rather than as direct temporal inferences from the fMRI signal.

Moreover, beyond cortico‐centric models of attention–emotion interactions, underscoring fronto‐parietal areas such as the FEF and the IPS (Corbetta et al. 2000), recent work has highlighted the contribution of subcortical hub structures, including the superior colliculus and the pulvinar, also implicated in integrating sensory, attentional, and socio‐emotional signals (Saalmann et al. 2012; Bourgeois et al. 2020). In particular, recent in vivo parcellation of the human superior colliculus has revealed distinct subdivisions with differential connectivity to both cortical attention networks and limbic regions, including the amygdala (Diano et al. 2025). This organization supports the view that emotional processing may rely on parallel and partially independent routes, which can remain functional even when top‐down cortical control is perturbed. Within this framework, the preserved or enhanced amygdala responses observed after FEF perturbation in our study do not necessarily imply a direct causal pathway from the FEF to the amygdala, but accord with the engagement of alternative subcortical routes that may be less dependent on voluntary attentional control.

Finally, we note that although behavioral modulations by emotion following rTMS were observed selectively in the FEF group at the within‐group level, direct between‐group comparisons of TMS‐induced changes in RTs did not reach statistical significance. This indicates that the present behavioral data alone do not allow a definitive conclusion regarding stimulation‐site specificity. Importantly, however, the converging group‐specific effects observed in fMRI results further support stimulation‐site–dependent effects on neural modulations in the fusiform cortex and amygdala following TMS to the FEF, but not the vertex, in agreement with a priori hypotheses concerning the functional role of attentional circuits in neural control of visual and emotional processing at the neural level.

### 4.2 Control Condition (Vertex Group)

In sharp contrast with the above, results from the control group receiving rTMS over the vertex (VTX) indicated no significant modulation by neurostimulation in any of the key ROIs implicated in our visual task. In this group, behavioral performance was similar to the FEF group prior to rTMS, with slower RTs when matching faces than when matching houses, but this did not change after TMS over the VTX, unlike the FEF group. These data reinforce the validity of our stimuli and experimental paradigm. Likewise, fMRI analyses in the VTX group confirmed the expected attentional and lateralization effects in the FFA and the PPA, but showed no modulation by TMS in any condition, clearly supporting the specific role of the FEF in the attentional and emotional modulation of facial processing observed in the experimental group. The amygdala exhibited significant interactions of Emotion with Hemisphere and Attention, but again no effect of TMS stimulation. Thus, in this group, amygdala responses to fearful faces were partly shaped by the task‐driven attentional modulation, while remaining unaffected by non‐specific cortical inhibition unrelated to attentional control.

### 4.3 Implication for Attention‐Emotion Interactions

Taken together, our results shed new light on the dynamic relationships between attentional and emotional systems, and provide important insights into how the FEF participates in the regulation of both cortical and subcortical (amygdala) responses to emotional salience.

These findings may also help explain some discrepancies reported in previous studies on attention–emotion interactions, in line with the framework proposed by Diano et al. (2016, 2017a, 2017b) according to which distinct patterns may arise in the visual cortex and the amygdala depending on differences in task design and attentional demands across studies. Evidence for relatively attention‐independent processing of emotion signals in the amygdala (e.g., Vuilleumier et al. 2001; Williams et al. 2004; Whalen et al. 1998) or instead attention‐gated responses in both the amygdala and the cortex (e.g., Pessoa, Kastner, and Ungerleider 2002; Pessoa et al. 2003) might be obtained due to methodological differences in the paradigms and measures used. In particular, the experimental manipulation of attention control may variably disrupt top‐down modulation of visual cortical responses or inhibitory regulation of amygdala reactivity. Along these lines, block paradigms might be more susceptible to habituation and to phasic prefrontal activation due to task predictability (Malsert et al. 2012), which might attenuate more automatic emotional interference effects. In contrast, event‐related paradigms (as used here) might favor tonic modulation of attention and emotion due to stronger and sustained top‐down engagement (Malsert et al. 2012; Malsert and Grandjean 2016). Accordingly, such variability and dynamic recruitment (e.g., prefrontal) mechanisms of cognitive control could determine different patterns of interactions between attentional and emotional systems, permitting emotional interference to emerge primarily in contexts of low stimulus predictability or less focused attentional engagement—a pattern consistent with the adaptive function of threat detection in naturalistic conditions. Importantly, the present findings do not allow us to disentangle the precise anatomical routes mediating these effects, nor to establish the temporal precedence of cortical versus subcortical contributions. Rather, they provide evidence that disrupting a key node of the dorsal attention network differentially constrains cortical and amygdala responses to emotional stimuli, in a manner consistent with—but not directly demonstrative of—parallel cortical and subcortical processing routes.

## 5 Conclusion

In sum, our observations suggest that inhibitory perturbation of the right FEF does not suppress the influence of emotional signals on face processing in the visual cortex, but may be associated with a relative preservation or enhancement of subcortical emotional responses under conditions of reduced attentional control. Consequently, the processing of emotionally salient stimuli seems relatively preserved—or even enhanced—under conditions of attentional disruption after rTMS. This mechanism is consistent with neuropsychological findings in patients with spatial neglect, who exhibit intact emotional biases in attentional tasks (faces: Lucas and Vuilleumier 2008; voices: Grandjean et al. 2008), often with larger effects than those observed in healthy participants. More generally, our new data accord with a two‐stage framework of emotion processing (Diano et al. 2016, 2017b; Vuilleumier 2005; Schettino et al. 2012). In this view, top‐down control processes mediated by the FEF may act not only on the visual cortex to regulate selective perceptual processing, enhancing relevant sensory inputs and suppressing irrelevant inputs, but also implement emotion regulation mechanisms over the amygdala to minimize involuntary or “automatic” responses to emotional information that could impede task performance or interfere with goals (McRae et al. 2010). Rather than supporting a strict dependence of emotion on attention, this framework points toward parallel and distinct influences of both systems—enabling engagement in specific task‐related goals through attentional regulation, while preserving the capacity to detect salient and potentially threatening stimuli when they occur unexpectedly.

## 6 Limitations

Despite the strengths of the present study—including the use of a within‐subject design, a concurrent TMS‐fMRI protocol, and the orthogonal manipulation of attention and emotion—some limitations should be acknowledged. First, the absence of significant emotional effects in the NoTMS (vertex) condition may reflect a potential ceiling effect, particularly in highly attentive participants, which could have masked subtle emotional modulations under baseline conditions. Second, although eye position was monitored online during scanning, the quality of the eye‐tracking recordings did not allow reliable offline analyses. As a result, gaze position could not be quantitatively assessed, and we cannot fully exclude subtle differences in gaze behavior across conditions. However, the brief stimulus presentation and task constraints strongly encouraged central fixation throughout the task, as previously reported for this paradigm (Vuilleumier et al. 2001). Third, the relatively small sample size may reduce the generalizability of the findings and limit the sensitivity of the task to subtle interaction effects, especially in subcortical structures like the amygdala. Fourth, although continuous theta‐burst stimulation (cTBS) is assumed to induce transient inhibition of the targeted cortical region, individual variability in neurophysiological responsiveness to TMS may introduce uncontrolled variance. This may also have contributed to (marginal) differences between the two groups. Finally, only fearful facial expressions were used to induce emotional salience. While fear is a well‐established signal of threat, future studies should investigate whether similar mechanisms apply to other negative (e.g., anger, disgust) or positive emotions.

## Supporting information


**Figure S1:** Amygdala responses in the vertex (VTX) group. Mean percent signal change (±1 SEM) extracted from spherical ROIs in the left and right amygdala for fearful and neutral faces, displayed for the NoTMS and TMS sessions and averaged across attention conditions. This figure illustrates the pattern of emotion‐ and hemisphere‐related modulations in the control group and allows direct comparison with the corresponding figure in the FEF group.

## Data Availability

The data that support the findings of this study are available from the corresponding author upon reasonable request.
