# Supplementary material for: Neural Substrates of Attentional Control During Emotional Processing: Evidence From rTMS–fMRI Targeting the Frontal Eye Field
Source: Hum Brain Mapp. 2026 Apr 28;47(7):e70535. doi: 10.1002/hbm.70535 (PMC13122430; doi:10.1002/hbm.70535)
Supplement: Supplementary file 1 — Figure S1: Amygdala responses in the vertex (VTX) group. Mean percent signal change (±1 SEM) extracted from spherical ROIs in the left and right amygdala for fearful and neutral faces, displayed for the NoTMS and TMS sessions and averaged across attention conditions. This figure illustrates the pattern of emotion‐ and hemisphere‐related modulations in the control group and allows direct comparison with the corresponding figure in the FEF group. [file HBM-47-e70535-s001.docx]

**SUPPLEMENTARY MATERIAL**


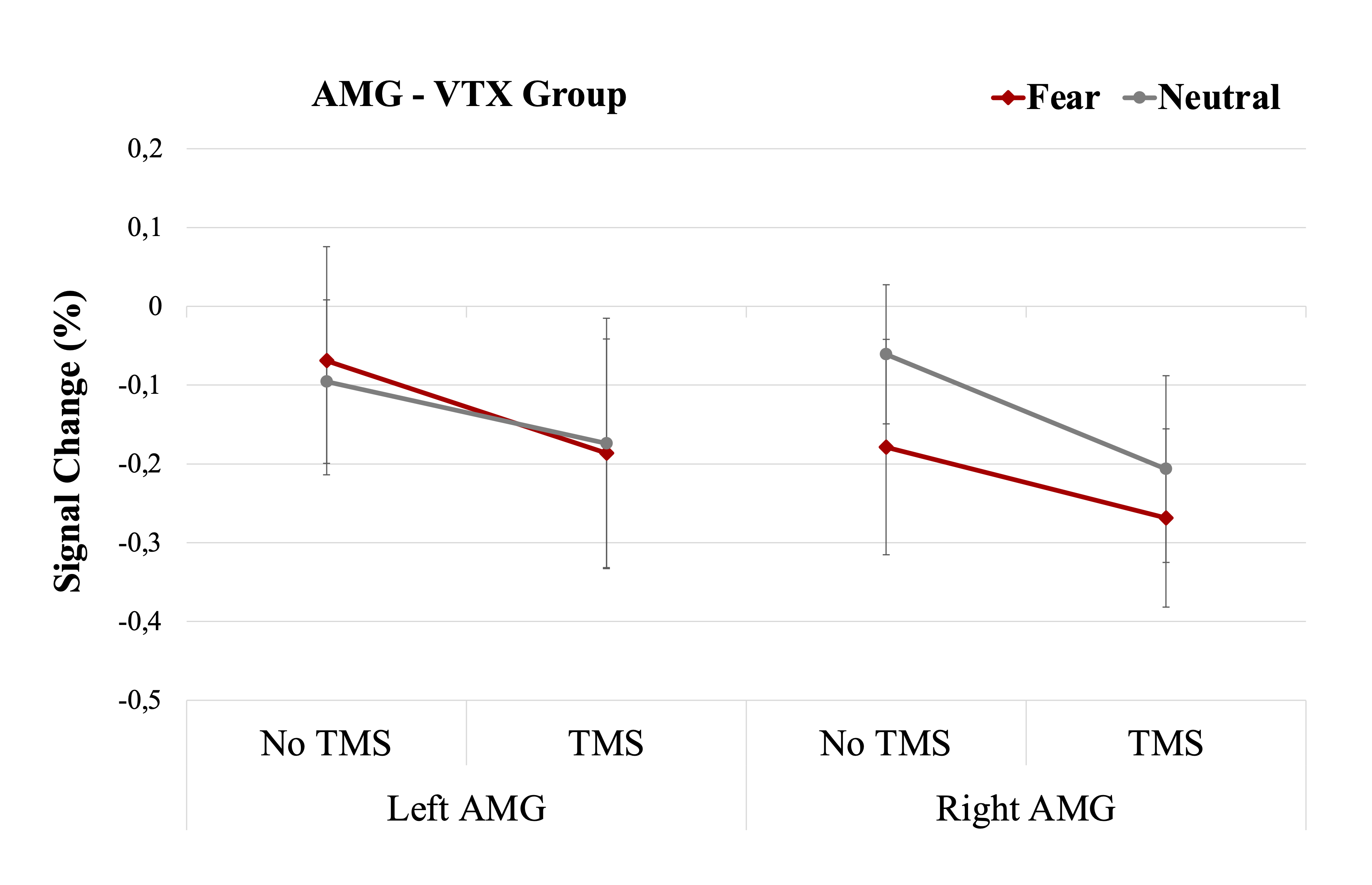


Figure S1. **Amygdala responses in the Vertex (VTX) group.** Mean percent signal change (±1 SEM) extracted from spherical ROIs in the left and right amygdala for fearful and neutral faces, displayed for the NoTMS and TMS sessions and averaged across attention conditions. This figure illustrates the pattern of emotion- and hemisphere-related modulations in the control group and allows direct comparison with the corresponding figure in the FEF group.
